# Infantile Convulsions

**Published:** 1891-03

**Authors:** 


Infantile Convulsions.—In this trouble Dr. A. Jacobi first administers a purgative dose of calomel. In a few hours this is followed by the following:

R Chloral hydrat., gr. iv.
Potas. bromid., gr. viij.
Aquæ,
Syrupi, aa ℥ j.

M. Sig. One dose for a child two years old.—*Ex.*

